# Acute Spinal Cord Ischemia Associated With Cocaine Use: A Case Report

**DOI:** 10.7759/cureus.25693

**Published:** 2022-06-06

**Authors:** Ramya Akella, Rishi Raj, Lakshmi Kannan, Aasems Jacob, Subramanya Shyam Ganti

**Affiliations:** 1 Internal Medicine, Pikeville Medical Center, Pikeville, USA; 2 Endocrinology, Diabetes and Metabolism, Pikeville Medical Center, Pikeville, USA; 3 Nephrology, Pikeville Medical Center, Pikeville, USA; 4 Hematology and Oncology, Pikeville Medical Center, Pikeville, USA; 5 Internal Medicine/Pulmonary Critical Care, Appalachian Regional Healthcare Internal Medicine Residency Program, Harlan, USA

**Keywords:** quadriplegia, spinal cord ischemia, myelopathy, cocaine, anterior spinal artery

## Abstract

Cocaine is one of the most common causes of acute drug-related emergency department visits in the United States. It produces a dose-dependent increase in heart rate and blood pressure accompanied by increased arousal and a sense of self-confidence, euphoria, and well-being. Its use is typically followed by a craving for more of the drug. It can also lead to acute events such as myocardial infarction, seizures, and cerebrovascular events. Here, we present a case of cocaine-induced spinal cord ischemia resulting in quadriplegia. Our case highlights that, in a young patient presenting with acute non-traumatic myelopathy, it is important to consider cocaine use among other differentials.

## Introduction

The use of recreational drugs has been increasing lately. The estimated global prevalence is 5%, with a prevalence of 19.4% among adults living in the United States [1]. Cocaine is one such recreational drug that acts as a stimulant. Recently, there has been a significant uptrend in cocaine use. According to the United Nations Annual World Drug Report 2021, cocaine is estimated to have been used by approximately 20 million people worldwide. A cross-sectional study showed that, since 2006, there has also been an increase in hospital admissions in the United States, associated with cocaine use, presenting with a variety of systemic manifestations. In that study, the total financial burden incurred due to cocaine use-related hospitalizations was around $10.8 billion in 2006 and increased to $19 billion by 2018. The impact of cocaine abuse on the healthcare system is a growing problem and there is a dire need to address this [2].

The vast majority of individuals who use cocaine use it infrequently and in small amounts. Routes of administration include intranasal, oral, and smoking. Spinal cord infarctions are relatively uncommon but can occur secondary to vascular surgery, severe hypotension, spine surgery, cardioembolic vertebral artery occlusion, and arteriovenous malformations. However, rarely, the use of cocaine can result in vasospasm of the spinal arteries leading to ischemic stroke. Because there are insufficient anastomoses for higher perfused regions in the upper cervical spinal cord levels, a loss of blood flow from the anterior spinal artery is hypothesized to induce anterior cord syndrome at the cervical level [1].

Furthermore, studies have shown that, at levels C2-C3 of the cervical spinal cord, there is an increased risk for ischemic events [3]. In some patients without clear etiologies, common vascular risk factors such as hypertension, diabetes, and cigarette smoking have been identified [3]. However, our patient did not have any of these common vascular risk factors, and cocaine use was the only identifiable risk, potentially contributing to the acute spinal cord ischemia.

## Case presentation

A 56-year-old white male presented to the emergency room with acute quadriplegia and neck pain that started about eight hours prior to the presentation. He woke up with neck pain and was unable to move bilateral upper and lower extremities. He also complained of decreased sensation from below his neck. He denied any recent head or neck trauma. A review of systems was negative for fever, chills, headache, and dysphagia. He had a medical history of post-ablative hypothyroidism. His social history was significant for use of recreational drugs (intravenous cocaine), daily cigarette smoking, and occasional use of alcohol. On physical examination, he had normal vital signs. Neurological examination revealed complete quadriplegia along with loss of sensation in all four extremities. Bilateral knee and ankle reflexes were absent, and he had poor rectal tone. There were no signs of meningeal irritation or tenderness in the cervical area, and examination of cranial nerves was completely normal. The rest of the examination was unremarkable except for needle marks on the left antecubital fossa.

**Figure 1:**
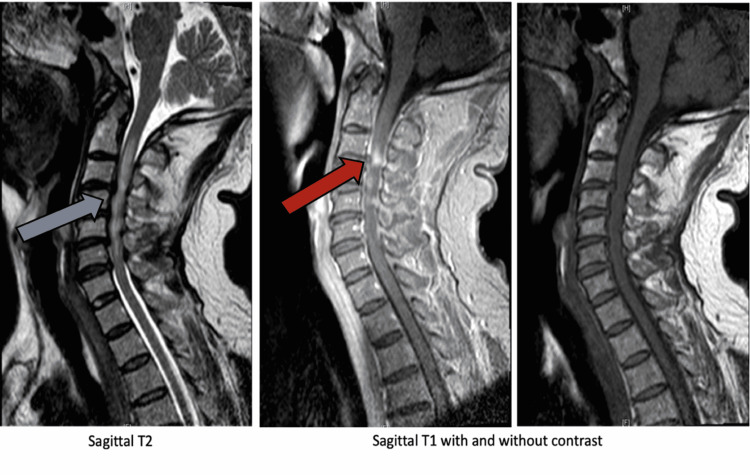
Magnetic resonance imaging of the cervical spine on presentation. Enhancement of the cord at C2 and C3 levels (red arrow) along with diffuse spinal cord edema between C2 and C7 (blue arrow).

Laboratory investigations were significant for positive urine drug screening for benzodiazepines and cocaine and mild azotemia (creatinine 1.5 mg/dL, blood urea nitrogen 22 mg/dL). The rest of the laboratory workup revealed normal hemogram, liver function tests, erythrocyte sedimentation rate, C-reactive protein), serum folate, and vitamin B12. Further testing for human immunodeficiency virus, rapid plasma reagin for syphilis, hepatitis B, and hepatitis C were negative. Non-contrast computed tomography (CT) scan of the head was negative. Magnetic resonance imaging (MRI) of the brain and spine revealed enhancement of the cord at C2 and C3 levels along with diffuse spinal cord edema between C2 and C7 (Figure 1).

The patient was admitted to the intensive care unit with a working diagnosis of acute cord ischemia versus transverse myelitis. He was urgently treated with one dose of intravenous methylprednisone 1 g, and neurology was consulted. Lumbar puncture was performed, and cerebrospinal fluid (CSF) was colorless and clear, with zero red blood cells, two white blood cells, glucose 74 mg/dL, protein 278 mg/dL, no oligoclonal bands, and negative cytology negative. As CSF analysis ruled out transverse myelitis and positive history of recent cocaine use prior to symptom onset, he was managed as a case of acute spinal cord ischemic stroke secondary to cocaine use. He was then continued on intravenous methylprednisolone 1,000 mg daily for five days. On the second day of hospital admission, the patient developed rapid shallow breathing and required mechanical ventilation. He had minimal recovery of motor and sensory function despite five days of high-dose steroids. His hospital course was complicated by severe gastrointestinal bleeding and ventilator-associated pneumonia resulting in a prolonged hospital stay. He underwent tracheostomy and percutaneous endoscopic gastrostomy tube placement, and was eventually discharged to a skilled nursing facility on hospital day 35.

## Discussion

In the context of active cocaine usage, our patient developed acute quadriplegia and workup revealed acute spinal cord ischemia at the cervical level, confirming myelopathy to be secondary to cocaine. Other causes of acute myelopathy include dural arteriovenous fistula, venous hypertension, tumors and infections of the spinal cord, and inflammatory conditions such as multiple sclerosis and transverse myelitis. In acute transverse myelitis, there is a clearly defined sensory level with features suggestive of inflammation within the spinal cord, such as CSF pleocytosis or elevated immunoglobulin (Ig)G index or gadolinium enhancement. Progression to nadir between four hours to twenty-one days following the onset of symptoms is another inclusion criterion for transverse myelitis. Our patient did not have elevated IgG or CSF pleocytosis.

Although cocaine usage has been linked to cerebrovascular events, there have been few reports of cocaine abuse-related acute spinal cord ischemia [1]. Stroke is more common in people using cocaine, while myelopathy is rare, with most cases describing the involvement of the anterior spinal cord. The ischemia tends to occur in the high to the mid-cervical region [1]. The current research indicates that young adults in the age range of 19-37 years are commonly involved, and in the majority, the lesion is between C2 and C7 in the anterior spinal artery territory of the cervical spinal cord. T1-T4 levels were involved in one case, while the posterior spinal artery was involved in another case at the cervical level (C2) [1]. Unfortunately, there are no distinguishing clinical or neuroradiological features to distinguish cocaine-induced myelopathy from more common causes of ischemic myelopathy [4]. Our case highlights that, in acute nontraumatic myelopathy, particularly in young patients, cocaine-induced ischemic myelopathy should be considered and ruled out in the differential diagnosis, even though it is rare. The mechanism of cocaine-induced spinal cord dysfunction is believed to be multifactorial and can be secondary to acute thrombosis, vasospasm [5], and possible vasculitis [6,7]. Research indicates that cocaine can induce platelet activation and aggregation in vitro and in vivo. Cocaine may block the reuptake of vasoconstrictors causing sympathetic overactivity, and cocaine itself can induce vascular inflammation. Severe neurological complications, such as quadriplegia and aphasia, can occur with the mucosal administration of cocaine, even in low doses [8].

When cocaine abuse causes quadriplegia and respiratory failure, complete recovery is rare. There is one case report with good recovery in which the patient had only transient vasospasm of the anterior spinal artery causing spinal cord ischemia [9]. In general, functional and neurological outcomes appear to depend on the American Spinal Injury Association impairment scale grade, level of the lesion, and (possibly) age [1].

Patients with acute spinal cord ischemia can develop neurogenic shock with hypotension and bradycardia. It is crucial to maintain adequate blood pressure to maintain adequate perfusion to the ischemic but not yet infarcted spinal cord. These patients are at high risk for respiratory failure requiring mechanical ventilation; hence, they should be closely monitored in the intensive care unit, with frequent neurological examinations. In select cases, high doses of steroids should be considered to decrease edema while pending definitive diagnosis.

## Conclusions

This case highlights that, even though it is rare, cocaine can cause acute myelopathy due to vascular ischemia. Moreover, there are no specific clinical or radiologic features that can accurately point to cocaine-induced myelopathy. Hence, for early diagnosis and appropriate management, physicians should have a high index of suspicion, especially in young patients presenting with acute non-traumatic myelopathy. The impact of cocaine abuse on the healthcare system is a growing problem, and addressing this issue appears to be the need of the hour.
